# A stem cell marker KLF5 regulates *CCAT1* via three-dimensional genome structure in colorectal cancer cells

**DOI:** 10.1038/s41416-021-01579-4

**Published:** 2021-10-27

**Authors:** Takashi Takeda, Yuhki Yokoyama, Hidekazu Takahashi, Daisuke Okuzaki, Kaho Asai, Hiroaki Itakura, Norikatsu Miyoshi, Shogo Kobayashi, Mamoru Uemura, Toshitsugu Fujita, Hiroo Ueno, Masaki Mori, Yuichiro Doki, Hodaka Fujii, Hidetoshi Eguchi, Hirofumi Yamamoto

**Affiliations:** 1 Department of Surgery, Gastroenterological Surgery, Graduate School of Medicine, Osaka University, 2-2, Yamadaoka, Suita, Osaka, 565-0871 Japan; 2 Department of Molecular Pathology, Division of Health Sciences, Graduate School of Medicine, Osaka University, 1-7, Yamadaoka, Suita, Osaka, 565-0871 Japan; 3 Single Cell Genomics, Human Immunology, WPI Immunology Frontier Research Center, Osaka University, 3-1, Yamadaoka, Suita, Osaka, 565-0871 Japan; 4 Department of Biochemistry and Genome Biology, Graduate School of Medicine, Hirosaki University, 5 Zaifu-cho, Hirosaki, Aomori, 036-8562 Japan; 5 Department of Stem Cell Pathology, Kansai Medical University, 2-5-1 Shin-machi, Hirakata, Osaka, 573-1010 Japan; 6 School of Medicine, Tokai University, 143 Shimokasuya, Isehara, Kanagawa 259-1193 Japan

**Keywords:** Colon cancer, Cancer genomics, Cancer stem cells

## Abstract

**Background:**

KLF5 plays a crucial role in stem cells of colorectum in cooperation with *Lgr5* gene. In this study, we aimed to explicate a regulatory mechanism of the *KLF5* gene product from a view of three-dimensional genome structure in colorectal cancer (CRC).

**Methods:**

In vitro engineered DNA-binding molecule-mediated chromatin immunoprecipitation (enChIP)-seq method was used to identify the regions that bind to the *KLF5* promoter.

**Results:**

We revealed that the *KLF5* promoter region interacted with the *KLF5* enhancer region as well as the transcription start site (TSS) region of the *Colon Cancer Associated Transcript 1 (CCAT1)* gene. Notably, the heterodeletion mutants of *KLF5* enhancer impaired the cancer stem-like properties of CRC cells. The KLF5 protein participated in the core-regulatory circuitry together with co-factors (BRD4, MED1, and RAD21), which constructs the three-dimensional genome structures consisting of *KLF5* promoter, enhancer and *CCAT1* TSS region. In vitro analysis indicated that KLF5 regulated *CCAT1* expression and we found that *CCAT1* expression was highly correlated with KLF5 expression in CRC clinical samples.

**Conclusions:**

Our data propose the mechanistic insight that the KLF5 protein constructs the core-regulatory circuitry with co-factors in the three-dimensional genome structure and coordinately regulates *KLF5* and *CCAT1* expression in CRC.

## Background

A zinc finger transcription factor Krüppel-like factor 5 (KLF5) plays an important role in the stemness of embryonic stem (ES) cells [1, 2] and developmental processes of respiratory epithelium, bladder epithelium, mammary gland and ocular surface [3–5]. In the intestinal epithelium, KLF5 is suggested to play a role in the maintenance of intestinal stem cells and their niche [6, 7].

*KLF5* has been reported as a lineage-survival oncogene whose expression is upregulated in specific cancer types, such as squamous carcinoma (e.g., head and neck cancer, oesophageal cancer) and gastrointestinal cancer (e.g., colorectal cancer, gastric cancer, pancreatic cancer) [8]. Cancer stem cells (CSCs) are defined as a subpopulation of cancer cells with a self-renewal capacity and multilineage potency and considered a source of tumour recurrence and metastasis [9]. KLF5 is associated with CSC-like properties because KLF5 knockdown suppressed sphere-formation activity in colorectal cancer (CRC) cell lines [10]. We also reported that miR-4711-5p, which directly targets the 3’-untranslated regions (3’-UTRs) of *KLF5*, suppressed CSC properties in CRC cell lines [11]. Moreover, *KLF5* deletion prevented the tumourigenesis of Lgr5^+^ intestinal stem cells induced by the mutated *β-catenin* gene [12].

Although the underlying mechanism of how the *KLF5* gene product is expressed in CRC has not been clarified, one of the mechanisms might be due to the three-dimensional genome structure of the *KLF5* gene. Cell-type-specific gene expression is occasionally regulated by 3D genome structure, as represented by promoter–enhancer looping. It is reported that promoter–enhancer looping takes place when cells undergo differentiation in developmental processes, leading to essential gene expression [13, 14]. In instances, the gene-activation element called the locus control region (LCR) interacts with the *γ-globin* gene in fetuses, and its interaction is switched to the *β-globin* gene in adults [15]. We also previously demonstrated that the CSC-related gene *ALDH1A1* was regulated by BRD4-dependent promoter–enhancer looping in ovarian cancer [16]. It is well known that the BRD4 as well as mediator complex and cohesin complex play a crucial role in the construction of 3D genome interaction [17]. MED1 and RAD21 are one of the components of the mediator complex and cohesin complex, respectively [18, 19].

Enhancers regulate gene expression by interacting with a promoter and are marked by histone modifications, such as acetylation at lysine 27 of histone H3 (H3K27ac) and monomethylation at lysine 4 of histone H3 (H3K4me1). In an effort to identify the *KLF5* enhancer region in CRC cells, we used in vitro engineered DNA-binding molecule-mediated chromatin immunoprecipitation (enChIP) combined with next-generation sequencing (NGS) (in vitro enChIP-seq) [20, 21]. In addition to the enhancer region, we found that the *KLF5* promoter region interacted with a long noncoding RNA, the *Colon Cancer Associated Transcript 1 (CCAT1)*. *CCAT1* is a long noncoding RNA that was initially found to be upregulated in CRC [22], and studies have reported that high *CCAT1* expression is associated with poor prognosis in CRC patients [23]. Subsequently, overexpression of *CCAT1* was reported in various cancer types, such as gastric cancer and oesophageal cancer [24–26]. *CCAT1* functions as a sponge for miRNAs [27], thereby contributing to malignant features of CRC by promoting cell proliferation, invasion and drug resistance [28, 29]. Finally, we show that the KLF5 protein constructs the core-regulatory circuitry with co-factors in the three-dimensional genome structure involving *KLF5* gene and *CCAT1*.

## Methods

### Cell lines and cell culture

Human CRC cell lines HT29 and SW48 were purchased from the American Type Culture Collection. Cells were cultured at 37 °C in 5% CO_2_. The mycoplasma test was performed prior to experiments. The KLF5 inhibitor ML264 [30] was purchased from Sigma Aldrich (St. Louis, MO, USA).

### Clinical tissue samples

Clinical tissue samples for RNA extraction were collected from CRC patients (*n* = 131) who underwent surgery at the Osaka University hospital from 2003 to 2005. For the immunohistochemistry and in situ hybridisation, tissue samples were collected from CRC patients (*n* = 27) who underwent surgery from April 2019 to June 2019. Informed consent signatures were obtained from all patients. This study was approved by the Ethics Committee of Osaka University Hospital (No. 15144).

### Real-time quantitative PCR (qRT-PCR)

The total RNA was extracted from cell lines and tissue samples by using the RNeasy Kit (QIAGEN, Hilden, Germany). qRT-PCR was performed as previously reported [11]. The sequences of the primers are listed in Supplementary Table S1.

### siRNA transfection

siRNAs for *BRD4* and negative control were purchased from GeneDesign (Osaka, Japan). siRNAs for *MED1*, *RAD21* and *KLF5* were purchased from Thermo Fisher Scientific (Waltham, MA, USA). siRNAs for *CCAT1* were purchased from GeneDesign and Thermo Fisher Scientific. siRNA (50 nM) was transfected into CRC cell lines 24 h after seeding with Lipofectamine RNAiMAX reagent (Thermo Fisher Scientific). For the triple-knockdown experiment, siRNAs (30 nM) for *BRD4*, *MED1* and *RAD21* were mixed and transfected into CRC cell lines. As a control, 90 nM of negative control (NC) siRNA was transfected into CRC cell lines. The sequences of the siRNAs are listed in Supplementary Table S1.

### Cell-proliferation assay

Cells were seeded in 96-well plates at a density of 4000 cells per well, and 5-FU or oxaliplatin was added 24 h after seeding. Three days later, cell viability was evaluated by using Cell Counting Kit-8 solution (Dojindo Laboratories, Kumamoto, Japan).

### Sphere-formation assay

Sphere-formation assay was performed as previously described [11]. At 3 weeks after seeding, the number of spheres (≥50 µm) in all wells was counted.

### Flow cytometry analysis

Cells were washed with PBS containing 2% FBS and incubated with primary antibody (Ab), anti-CD133/1 (AC133)-APC (Miltenyi Biotec, Bergisch Gladbach, Germany) or anti-CD44v9 (Cosmo Bio, Tokyo, Japan) at 4 °C for 20 min. For CD44v9, PE mouse anti-rat IgG2a (Becton, Dickinson and Company, Franklin Lakes, NJ, USA) was used as a secondary Ab. The data were analysed by using the SA3800 spectral cell analyser (Sony, Tokyo, Japan).

### Immunohistochemical staining

Immunohistochemical staining was performed as described previously [31]. Rabbit polyclonal Ab for KLF5 (Sigma Aldrich) was used as a primary Ab. All specimens were evaluated individually by four researchers.

### RNA scope^®^

Paraffin-embedded tumour tissue samples were sectioned into 4-µm sections and *CCAT1* signals were detected using the Hs-CCAT1 target probe (Advanced Cell Diagnostics, Newark, CA, USA) and subsequently stained with RNA scope^®^ [32] (Advanced Cell Diagnostics).

### ChIP-qPCR

ChIP experiment was performed as previously reported [33]. Cells were fixed and sheared by using Covaris M200 (Covaris, Woburn, MA, USA). The fragmented chromatin was incubated with the following primary Abs; KLF5 (Abcam, Cambridge, UK), MEIS1 (Abcam), RHOXF1 (GeneTex, Irvine, CA, USA), ZNF354C (Abcam), BRD4 (Bethyl Laboratories, Montgomery, TX, USA), MED1 (Bethyl Laboratories), RAD21 (Abcam). The Ab against normal rabbit IgG (Cell Signaling Technology, Danvers, MA, USA) was used as a negative control. The purified DNA was subjected to qPCR. qPCR was performed as described in the qRT-PCR section. The sequences of the primers are listed in Supplementary Table S1.

### Deletion of the genomic region by CRISPR/Cas9

The Cas9 expression plasmid, hCas9 (a gift from Dr. George Church, Addgene, #41815; http://n2t.net/addgene:41815; RRID:Addgene_41815) [34] gRNA Cloning Vector BbsI ver. 2 plasmid (Addgene, #85586; http://n2t.net/addgene:85586; RRID:Addgene_85586) [35] and pEGFP-N3 (Clontech Laboratories, Mountain View, CA, USA) were used for the experiment. The synthesised nucleotides were annealed and cloned into the gRNA Cloning Vector BbsI ver.2 plasmid.

HT29 cells were transfected with the hCas9 plasmid and two gRNA expression plasmids that target both ends of the *KLF5* enhancer or *CCAT1* TSS region, and pEGFP-N3 with Lipofectamine 3000 (Thermo Fisher Scientific). The next day, GFP-positive cells were sorted and seeded individually in 96-well plates.

### In vitro enChIP-seq and bioinformatics analysis

In vitro enChIP-seq was performed as previously reported [21]. Guide RNAs for the *KLF5* promoter (gRNA-A, B) were designed by using the CRISPRdirect Web tool (https://crispr.dbcls.jp/). As a negative control, gRNA, which is designed in the promoter region of the chicken *Pax5* gene, was used [21]. The crRNAs and tracrRNAs were synthesised by FASMAC (Kanagawa, Japan). The purified DNA was subjected to qPCR or NGS. The in vitro enChIP-seq library was prepared by using the TruSeq ChIP Library Preparation Kit (Illumina, San Diego, CA, USA). The libraries were sequenced as 36-bp single-end reads on the HiSeq3000 (Illumina). Images of NGS peaks were generated using Integrative Genomics Viewer (IGV) (http://software.broadinstitute.org/software/igv/). The sequences of the gRNAs and primers are listed in Supplementary Table S1.

### Statistical analysis

Data are shown as the mean ± SD. The data were compared by Student’s *t* test, chi-square test, or Fisher’s exact test. The Kaplan–Meier method and log-rank test were used to calculate significant differences in patient survival. A value of *P* < 0.05 was considered statistically significant. All statistical analyses were performed using Microsoft Excel or JMP statistical software (SAS Institute Inc., Cary, NC, USA).

## Results

### Identification of the enhancer region for the *KLF5* gene

To identify the enhancer region that binds to the *KLF5* promoter, we employed an in vitro enChIP-seq method [20] (Supplementary Fig. S1A). The binding DNA sequence was analysed by NGS using two guide RNAs (gRNA-A, gRNA-B) designed 400–600 bp upstream of the transcription start site in the *KLF5* promoter region (Supplementary Fig. S1B).

**Fig. 1:**
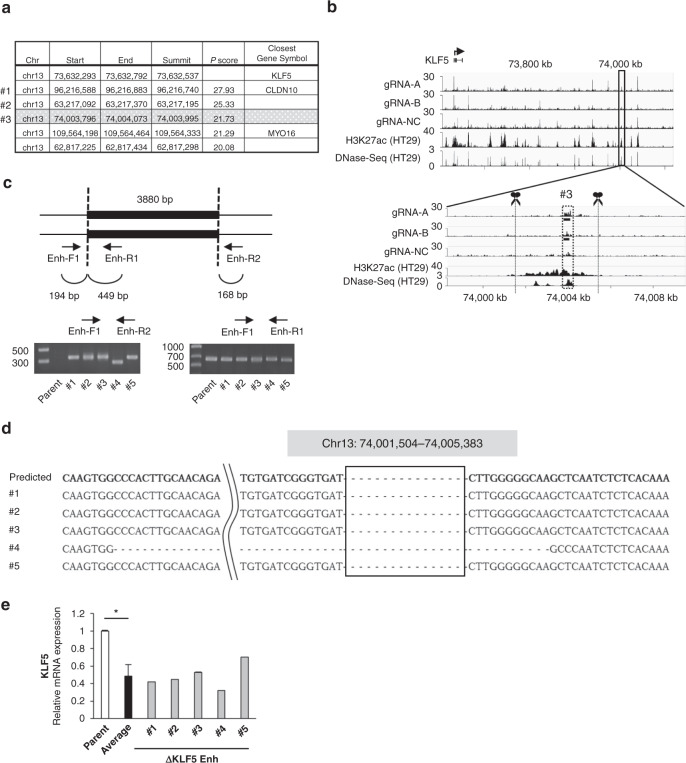
Identification of the *KLF5* enhancer region by in vitro enChIP-seq. **a** List of regions that bind to the *KLF5* promoter by in vitro enChIP-seq analysis. The regions of the top five highest *P* scores on chromosome 13 are shown. First line shows the *KLF5* promoter region including the target region of two gRNAs. The obtained reads from NGS were mapped to the human hg19 reference genome using the COBWeb algorithm. Peak calling was performed using the MACS peak detection algorithm at default settings on Strand NGS software version 3.4 (Agilent Technologies, Santa Clara, CA, USA). The peaks were determined using gRNA-A and gRNA-B as biological duplicates against gRNA-NC (negative control) filtered for *P* score (−log10(*P* values)) and fold change ≥2.0. **b** Integrative genome viewer (IGV) tracks of in vitro enChIP-seq peaks (gRNA-A, gRNA-B, gRNA-NC) and ChIP-seq peaks of H3K27ac and DNase-seq peaks from the ChIP-Atlas database in HT29 cells. The region surrounded by a dotted square is the rank #3rd peak listed in (**a**). Scissors indicate the position of the gRNAs to create the deletion mutants. **c** Schematic illustration of the *KLF5* enhancer candidate region deleted by the CRISPR/Cas9 system. The positions of the primers for validation are indicated. All candidate clones of the deletion mutant were validated by PCR. Gel images show the PCR product amplified by the indicated primers (Enh-F1 and Enh-R2 or Enh-F1 and Enh-R1) in parental cells and five heterodeletion mutants. **d** Sequencing result of PCR products that are amplified by Enh-F1 and Enh-R2 primers in five heterodeletion mutants. The deleted region is surrounded by a square and the predicted sequence after deletion is shown as ‘Predicted’. **e** Expression level of *KLF5* mRNA in five deletion mutants. The relative value is calculated by the expression level of the parental cells. The average value of five heterodeletion mutants is also shown. *P < 0.01.

Compared with the negative control gRNA against the chicken *Pax5* gene [21], several DNA sequences had a significant increase in binding to the gRNA-marked *KLF5* promoter regions. As candidates for the *KLF5* enhancer, we first searched the genomic regions on chromosome 13 where *KLF5* promoter is located (Fig. 1a and Supplementary Fig. S2A). Among them, we focused on Chr 13; 74,003,796 − 74,004,073 (*P* score rank #3rd, Fig. 1a) located a~370 kb downstream of the *KLF5* promoter rather than the other two regions with superior *P* scores (rank #1st and #2nd) because the peaks of well-established enhancer markers such as histone H3 lysine 27 acetylation (H3K27ac) and BRD4 were robustly observed at this region compared with #1st and #2nd regions by the ChIP-seq database analysis (http://chip-atlas.org/, Supplementary Fig. S2B). The region displayed a significantly higher peak with gRNA-A or gRNA-B than the negative control gRNA, and it well matched the peak of the enhancer marker H3K27ac or DNase I hypersensitive site sequencing (DNase-seq), which is a marker for the open chromatin region [36] (Fig. 1b). To investigate whether this region would function as an enhancer, we generated five heterodeletion mutants of potential enhancer regions (Fig. 1c, d). All mutants showed decreased *KLF5* expression, and the average (51.7%) was significantly lower than that of parental cells (Fig. 1e, *P* < 0.01), indicating that the identified region functions as an enhancer of *KLF5* gene.

### *KLF5* promoter–enhancer looping is organised by KLF5 protein and co-factors

**Fig. 2:**
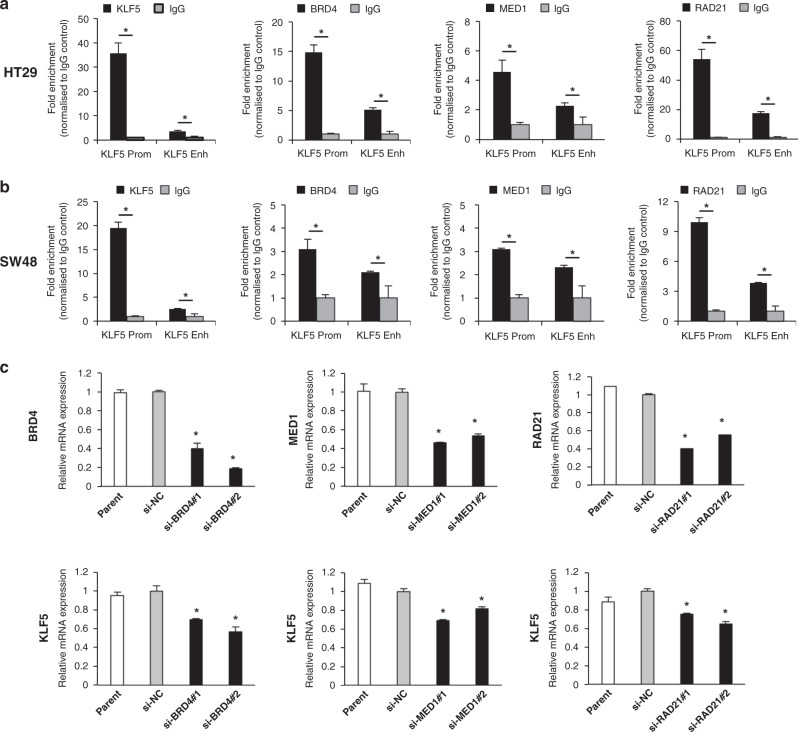
KLF5 protein and co-factors bind to the *KLF5* promoter and enhancer. **a**, **b** The binding of the KLF5, BRD4, MED1 and RAD21 proteins to the *KLF5* promoter and enhancer in HT29 (**a**) and SW48 (**b**) cell lines analysed by ChIP-qPCR. Fold enrichment normalised to the value of the IgG control is shown. *P < 0.01. **c** Expression level of *KLF5* mRNA in HT29 cells transfected with siRNAs against *BRD4*, *MED1* and *RAD21*. The relative value is calculated by the expression level of the si-negative control transfected cells (siNC). *P < 0.01.

We hypothesised that the KLF5 protein might bind to the *KLF5* promoter and/or the enhancer because ChIP-seq database showed this possibility in the KATO III gastric cancer cell line (http://chip-atlas.org/, Supplementary Fig. S3A). In addition, the JASPAR transcription–prediction tool [37] indicated that the transcription factor KLF5 protein is able to bind to both promoter and enhancer of *KLF5* gene (Supplementary Fig. S3B, C). ChIP-qPCR analyses revealed that the KLF5 protein bound to the *KLF5* promoter and enhancer in the HT29 and SW48 cell lines (Fig. 2a, b). In addition, we found that BRD4, MED1 (mediator complex protein) and RAD21 (cohesin complex protein) were also involved in the machinery constructing promoter–enhancer looping by binding to both regions (Fig. 2a, b). Moreover, a single knockdown of *BRD4*, *MED1* and *RAD21* and triple knockdown of these genes led to a modest decrease in *KLF5* expression (Fig. 2c and Supplementary Fig. S3D). These results suggest that the KLF5 protein and co-factors may contribute to construct the promoter–enhancer looping of the *KLF5* gene. Besides KLF5 protein, JASPAR transcription–prediction tool indicated that the transcription factors, Myeloid ecotropic viral integration site 1 (MEIS1), Reproductive homeobox on X-chromosome F1 (RHOXF1), Zinc finger protein 354 C (ZNF354C) proteins might have the potential to bind to both promoter and enhancer of *KLF5* gene (Supplementary Fig. S3B, C). Therefore, we performed ChIP-qPCR analyses and revealed that the MEIS1, RHOXF1 proteins, but not ZNF354C bound to both promoter and enhancer of *KLF5* gene (Supplementary Fig. S3E).

### The *KLF5* enhancer is associated with cancer stem-like properties in CRC cells

**Fig. 3:**
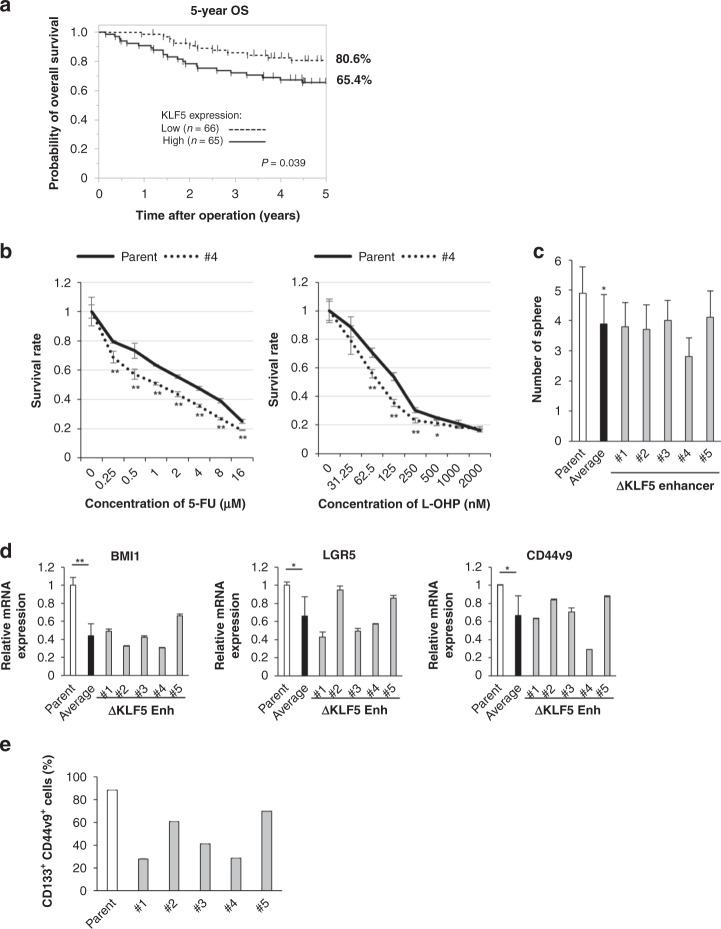
The deletion mutants of the *KLF5* enhancer show decreased cancer stem-like properties. **a** Kaplan–Meier survival analysis of overall survival for 131 CRC clinical samples classified by *KLF5* expression score. The high KLF5 expression group had poorer survival when compared with the low KLF5 expression group (*P* = 0.039). When the cut-off point was set at a median value of *KLF5* mRNA, there were no significant differences in patients’ backgrounds between the high-*KLF5* expression group (*n* = 65) and the low-*KLF5* expression group (*n* = 66) (Supplementary Table S2). **b** The survival rate of parental cells and heterodeletion mutant clone #4 of the *KLF5* enhancer after 5-FU and L-OHP treatment. *P < 0.05, **P < 0.01. **c** The number of spheres in parental cells and five heterodeletion mutants of the *KLF5* enhancer. Five hundred cells were seeded, and the number was counted 3 weeks after seeding. The spheres larger than 50 µm were counted. The average number of five heterodeletion mutants is also shown. *P < 0.01. **d** The expression level of CSC-related genes in parental cells and five heterodeletion mutants of the *KLF5* enhancer. The relative value is calculated by the expression level of the parental cells. The average value of five heterodeletion mutants is also shown. *P < 0.05, **P < 0.01. **e** The expression levels of CD44v9 and CD133 were analysed by flow cytometry in parental cells and five heterodeletion mutants of the *KLF5* enhancer. The percentage of double-positive cells are shown.

In human clinical CRC tissues (*n* = 131) we found that the high *KLF5* mRNA expression group had a worse prognosis than low *KLF5* mRNA expression group when the cut-off point was set at a median value of *KLF5* mRNA expression (*P* = 0.039, Fig. 3a and Supplementary Table S2). To investigate the functional relevance of the *KLF5* enhancer in cancer stem-like properties, we examined the chemoresistance, sphere-formation ability and expression level of CSC-related genes in heterodeletion mutants of the *KLF5* enhancer. The heterodeletion mutants were more sensitive to 5-FU and oxaliplatin (L-OHP) and showed lower sphere-formation activity than parental cells (Fig. 3b, c and Supplementary Fig. S4A, B). The cells had significantly decreased mRNA expression of the CSC-related markers *BMI1*, *LGR5* and *CD44v9* by RT-PCR (Fig. 3d), and the double-positive fractions of CD133 and CD44v9 in heterodeletion mutants also decreased to various extents compared with parental cells by flow cytometric analysis (parent 88.2% vs mutants 28.0, 61.0, 41.3, 28.7 and 69.7%) (Fig. 3e and Supplementary Fig. S4C).

### The *KLF5* promoter interacts with the *CCAT1* TSS region located on a distinct chromosome

**Fig. 4:**
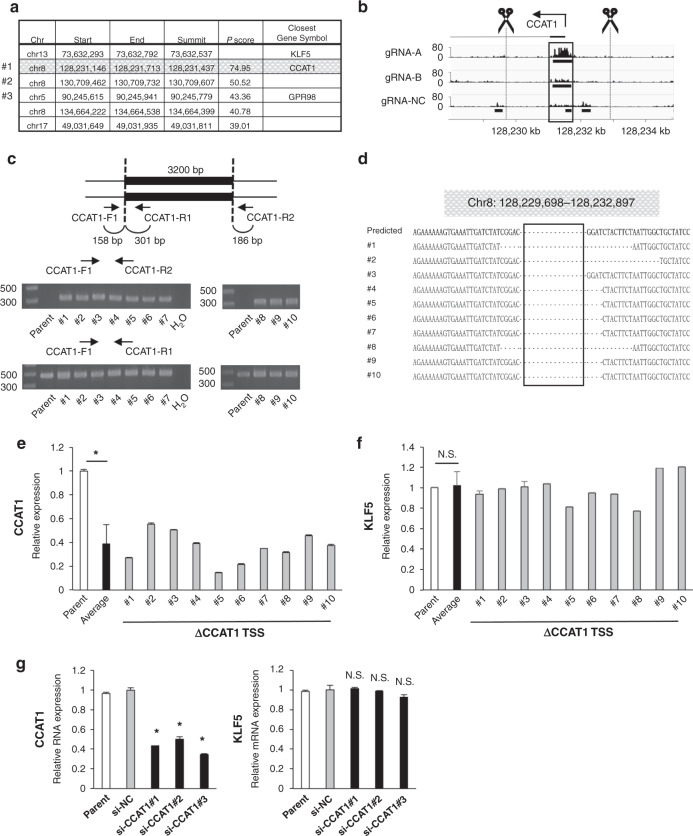
The *KLF5* promoter binds to the *CCAT1* TSS region. **a** List of regions that bind to the *KLF5* promoter by analysing the in vitro enChIP-seq data. The regions of the top five highest *P* scores in all chromosomes are shown. First line shows the *KLF5* promoter region including the target region of two gRNAs. **b** IGV track of the *CCAT1* TSS region. Scissors indicate the position of the gRNAs for creating the deletion mutants. The region surrounded by a square is the #1st peak. **c** Schematic illustration of the *CCAT1* TSS region deleted by the CRISPR/Cas9 system. The positions of the primers for validation are indicated. All candidate clones of the deletion mutant were validated by PCR. Gel images show the PCR product amplified by the indicated primers (CCAT1-F1 and CCAT1-R2 or CCAT1-F1 and CCAT1-R1) in parental cells and ten heterodeletion mutants. **d** Sequencing results of PCR products amplified by CCAT1-F1 and CCAT1-R2 primers in ten heterodeletion mutants. The deleted region is indicated as a square and the predicted sequence after deletion is shown as ‘Predicted’. **e** Expression level of *CCAT1* RNA in ten heterodeletion mutants of the *CCAT1* TSS region. The relative value is calculated by the expression level of the parental cells. The average value of ten heterodeletion mutants is also shown. *P < 0.01. **f** Expression level of *KLF5* mRNA in ten heterodeletion mutants of the *CCAT1* TSS region. The relative value is calculated by the expression level of the parental cells. The average value of ten heterodeletion mutants is also shown. N.S.   not significant. **g** Expression levels of *CCAT1* RNA and *KLF5* mRNA in *CCAT1* knockdown cells. The relative value is calculated by the expression level of the si-negative control transfected cells (siNC). *P < 0.01. N.S.  not significant.

We next analysed the interchromosomal interaction with the *KLF5* promoter. Among many candidates, the most significant interaction was found with the *CCAT1* TSS region located at chromosome 8 (Fig. 4a and Supplementary Fig. S5A). To clarify whether the *CCAT1* TSS region functions as an enhancer of the *KLF5* gene, we produced ten heterodeletion mutants of the *CCAT1* TSS region (Fig. 4b–d). We found that these mutants clones displayed a large reduction in *CCAT1* expression (Fig. 4e), but *KLF5* expression levels were not affected (Fig. 4f). At the transcription level, knockdown of *CCAT1* RNA did not affect *KLF5* expression either (Fig. 4g).

**Fig. 5:**
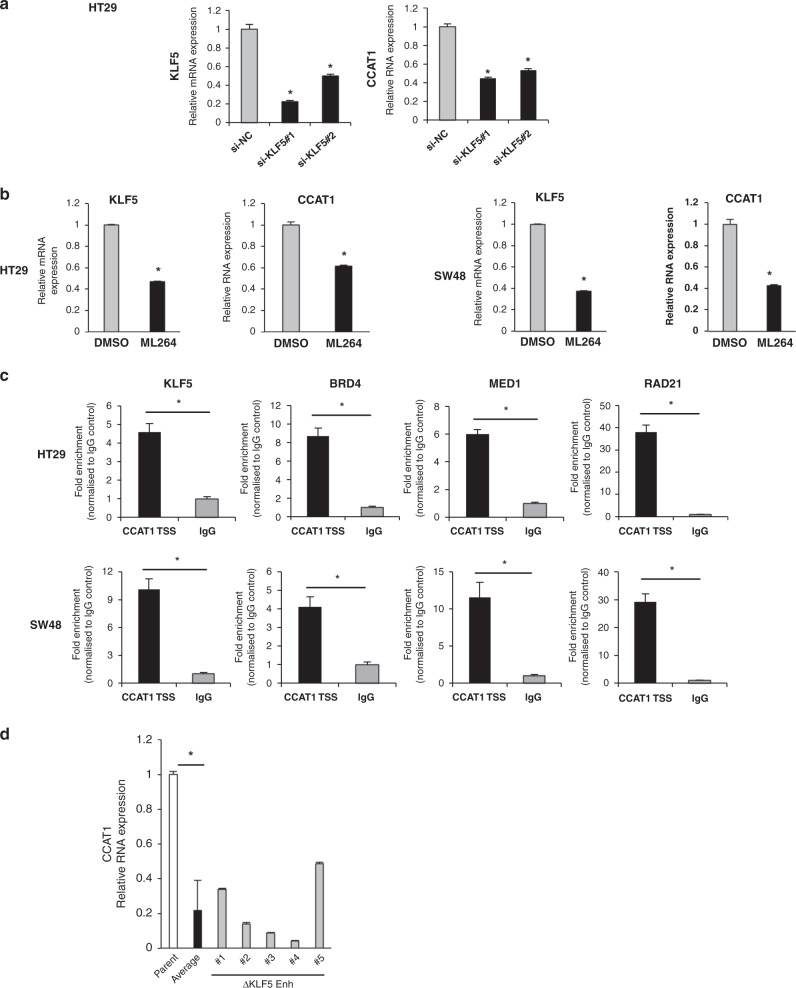
KLF5 protein regulates *CCAT1* expression. **a** Expression levels of *CCAT1* RNA and *KLF5* mRNA in *KLF5* knockdown cells. The relative value is calculated by the expression level of the si-negative control transfected cells (siNC). *P < 0.01. **b** Expression levels of *KLF5* mRNA and *CCAT1* RNA in ML264-treated cells (HT29, SW48). ML264 (10 µM) was treated for 24 h. The relative value is calculated by the expression level of DMSO-treated control cells. *P < 0.01. **c** The binding of the KLF5, BRD4, MED1 and RAD21 proteins to the *CCAT1* TSS region in the HT29 (top) and SW48 (bottom) cell lines analysed by ChIP-qPCR. The fold enrichment normalised to the value of the IgG control is shown. *P < 0.01. **d** Expression level of *CCAT1* RNA in five heterodeletion mutants of the *KLF5* enhancer. The relative value is calculated by the expression level of the parental cells. The average value of five heterodeletion mutants is also shown. *P < 0.01.

We next investigated the possibility that the *KLF5* gene product might conversely regulate *CCAT1* expression and found that knockdown of *KLF5* mRNA decreased *CCAT1* expression in HT29 cells (Fig. 5a), and a KLF5 inhibitor, ML264, also suppressed *CCAT1* expression in HT29 and SW48 cell lines (Fig. 5b). As a ChIP-seq database survey and a prediction of transcription factor-binding site showed that the KLF5 protein-binding region coincided with the *CCAT1* TSS region (Supplementary Fig. S5B and C), our ChIP-qPCR analyses revealed that the KLF5 protein and BRD4, MED1 and RAD21 bound to the *CCAT1* TSS region in the HT29 and SW48 cell lines (Fig. 5c). In heterodeletion mutants of the *KLF5* enhancer, we found that *CCAT1* expression markedly decreased, with an average of 21.8% (Fig. 5d, *P* < 0.01). Moreover, *BRD4*, *MED1* and *RAD21* knockdown significantly decreased the *CCAT1* expression (Supplementary Fig. S5D). These findings imply that the KLF5 protein and co-factors could participate also in the three-dimensional genome binding between *KLF5* gene and *CCAT1*.

### KLF5 and CCAT1 expression are correlated in CRC clinical samples

**Fig. 6:**
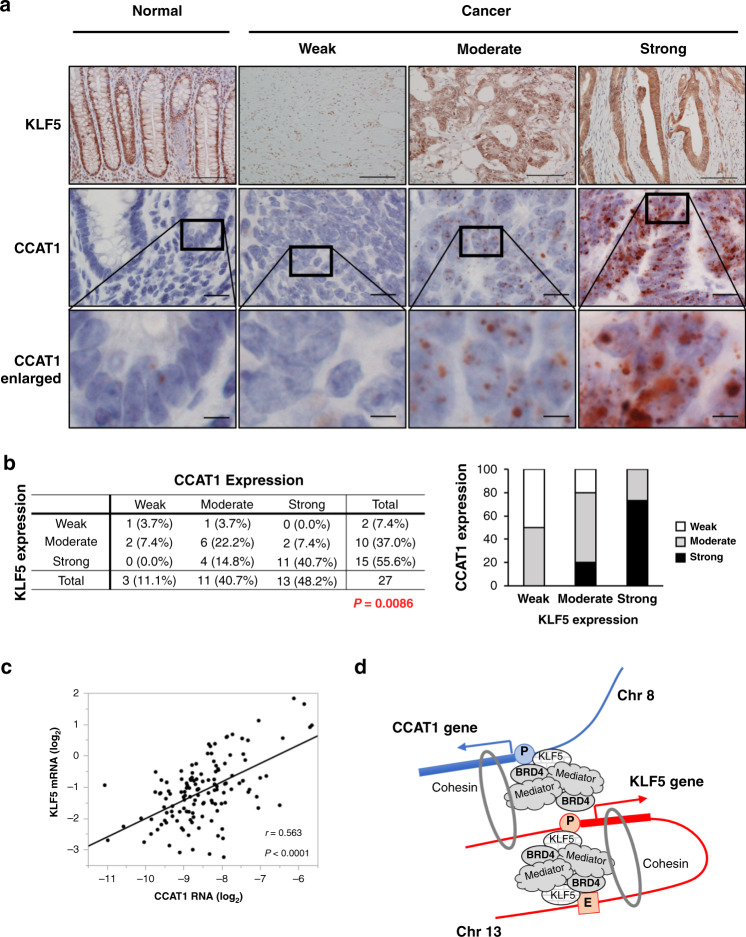
KLF5 and *CCAT1* expression is correlated in CRC tissues. **a** Representative images of KLF5 protein and *CCAT1* RNA expression in normal colorectal epithelium and CRC. The expression level of KLF5 in tumour cells was evaluated at ×100 magnification. Nuclear staining of the KLF5 protein was scored based on the percentage of positive cells as follows: weak: 0–10%, moderate: 11–50%, strong: 51–100%. The expression level of *CCAT1* in tumour cells was evaluated at ×100 magnification and scored as previously reported [32]. Score 0 was defined as weak, score 1–2 was defined as moderate, and score 3–4 was defined as strong. The bottom panels show the higher magnification image of the framed area. Scale bar: 100 μm (top), 20 μm (middle), 5 µm (bottom). **b** The percentage of each score (weak, moderate, strong) of KLF5 protein and *CCAT1* RNA expression in CRC clinical samples. The bar chart is also shown. **c** The correlation of *KLF5* mRNA and *CCAT1* RNA expression in 131 CRC clinical samples. The expression levels of *KLF5* mRNA and *CCAT1* RNA were significantly correlated (r = 0.563, *P* < 0.0001). **d** Illustration model showing the gene regulation mechanism for *KLF5* and *CCAT1* via the three-dimensional genome structure.

We next examined the expression of the KLF5 protein and *CCAT1* RNA in 27 CRC tissue samples by immunohistochemistry and in situ hybridisation using RNA scope [32]. In normal tissue, nuclear KLF5 expression was observed predominantly at the colonic crypt bottom as previously reported [12] (Fig. 6a). In cancer tissues, KLF5 expression was observed in the nucleus or cytoplasm, and CRC cancer samples were divided into three groups based on the nuclear staining as follows: weak: 7.4%, moderate: 37.0%, and strong: 55.6% (Fig. 6a, b). On the other hand, *CCAT1* was rarely expressed in normal epithelium and increased in cancer tissue (weak: 11.1%, moderate: 40.7%, strong: 48.2%) (Fig. 6a, b). The KLF5 expression score was significantly correlated with that of *CCAT1* (*P* = 0.0086, Fig. 6b; scoring system for KLF5 and *CCAT1* is described in the legend of Fig. 6a). This correlation was also observed when we analysed in well-differentiated adenocarcinoma samples (*P* = 0.0256, Supplementary Fig. S6). In addition, qPCR analysis using RNA extracted from 131 CRC patients indicated that the expression levels of *KLF5* mRNA and *CCAT1* RNA were significantly correlated (*r* = 0.563, *P* < 0.0001) (Fig. 6c).

## Discussion

It is recently reported that *KLF5* transcripts are actively regulated by three-dimensional structural changes through promoter–enhancer binding in head and neck cancer cells [38]. We investigated this possibility in CRC because the enhancer markers H3K27ac and BRD4 showed similar peaks downstream of the *KLF5* gene in HT29 colon cancer cells by a ChIP-seq database search (ChIP-Atlas: https://chip-atlas.org/, Supplementary Fig. S7A). As result, we found that the *KLF5* promoter bound not only to its enhancer region but also to lncRNA *CCAT1*.

Three-dimensional genome structure has been gradually uncovered due to the development of technologies such as Hi-C, chromatin interaction analysis by paired-end tag sequencing (ChIA-PET) analysis, and in vitro enChIP-seq method [20, 21, 39, 40]. Promoter– enhancer looping is constructed by the protein complex, including BRD4, the mediator complex, the cohesion complex and transcription factors [41, 42]. Using the in vitro enChIP-seq method, we identified a tightly bound region ~370 kb downstream of the *KLF5* gene (chr13, 74,003,796–74,004,073, 278 bp), and experiments using heterodeletion mutants of the enhancer verified that this region was indeed an enhancer of the *KLF5* gene. Although this region partially overlapped with one of the enhancer regions reported in head and neck cancer (chr13, 74,002,153–74,004,229, 2077 bp) [38], we successfully encompassed the essential region. During the cloning process, we eventually obtained only five heterodeletion mutants and no homodeletion mutants out of 672 single cells into which the CRISPR-Cas9 system was transduced. One possible reason for missing the homodeletion clones is that the *KLF5* gene is essential for cell survival. To support this idea, it was reported that *KLF5* homozygous knockout mice died before embryonic day 8.5 [43], and even mice with conditional complete deletion of *KLF5* in the gut died shortly after birth due to the absence of proliferation in the intestinal epithelium [44].

Cumulative evidence suggests that enhancer regions typically contain TF-binding motifs [36]^.^ For example, in murine embryonic stem cells, various enhancers are occupied by multiple TFs, including Oct4, Sox2 and Nanog [17]. Accordingly, we sought the TF-binding motif in the *KLF5* promoter and enhancer by the JASPAR tool to further elucidate the molecular mechanism of *KLF5* gene regulation. Notably, we found that the KLF5 protein itself was the candidate TF for regulating *KLF5* expression and confirmed binding of the KLF5 protein to not only the enhancer region but also the promoter together with co-factors including BRD4, MED1 and RAD21 by ChIP-qPCR. A similar instance was recently reported in oesophageal squamous cell carcinoma cell lines; KLF5 participated in the core-regulatory circuitry (together with TFs, TP63 and SOX2 and co-factors) to construct the three-dimensional genome of the *ALDH3A1* or *EGFR* gene [45]. Our finding may be more unique regarding autoactivation because the regulatory circuitry containing the KLF5 protein contributes to the three-dimensional genome structure of the *KLF5* gene. On the other hand, we found that other two transcription factors, MEIS1 and RHOXF1, also bound to both *KLF5* promoter and enhancer region, despite the binding may be weaker compared to the KLF5 protein. MEIS1 belongs to a family of the three amino acid loop extension (TALE) homeodomain transcription factor and it was reported that MEIS1 functions as the regulator of the cell cycle, cell proliferation and differentiation [46]. Of note, it was reported that MEIS1 is involved in superenhancer associated gene expression in combination with EWS-FLI in Ewing sarcoma [47]. RHOXF1 (originally called as OTEX and hPEPP1) is a member of Rhox gene family, which is expressed in ovary, testis, epididymis, prostate and mammary gland [48] and malignant diseases of prostate cancer, leukaemia and CRC [49, 50]. Further studies will be needed on whether these proteins are also involved in the core-regulatory circuitry.

Long-range genome interactions, as well as local genome interactions, are known to regulate cell-type-specific gene expression and maintain cell identity. Interchromosomal interactions have been demonstrated between *SOX9* and the lncRNA *CISTR-ACT* gene or the *ATF4* and *FIRRE* genes to serve biological processes including mammalian development and differentiation, as well as cancer stemness [51]. For the interchromosomal interaction, we focused on the specific binding between the *KLF5* promoter and the *CCAT1* TSS region because the peak of this region was even higher and more significant than that of intrachromasomal interaction including *KLF5* promoter–enhancer interaction (Figs. 1a, b and  4a, b).

Studies have shown that the *CCAT1* genomic region is a part of the superenhancer for the *MYC* gene and that *CCAT1* RNA facilitates *EGFR* expression through activation of the *EGFR* enhancer by recruiting the transcription factors p63 and SOX2 [52]. However, in the current case, the *KLF5* expression level was not altered when *CCAT1* DNA was heterogeneously deleted or the CCAT1 transcript was suppressed by the specific siRNAs. Conversely, *KLF5* knockdown by specific siRNAs and treatment with the KLF5 inhibitor suppressed *CCAT1* expression. Since the KLF5 protein bound to the *CCAT1* TSS region as well as the *KLF5* promoter and enhancer together with co-factors (BRD4, MED1, RAD21), we speculate that the *KLF5* gene product participates in the core-regulatory circuitry and may regulate *CCAT1* expression. We performed the knockdown experiment by treating the single or triple combination of siRNAs against co-factors (BRD4, mediator, cohesin) and the result showed that knockdown efficiency was sufficient in both conditions but the downregulation of *KLF5* was still modest. These results suggest that knockdown of co-factors alone was not enough to achieve the complete disruption of the regulatory machinery for *KLF5* gene expression although it is indeed partially involved. It is of interest, according to the CCLE gene expression database (https://portals.broadinstitute.org/ccle), that both *CCAT1* and *KLF5* RNAs are upregulated in gastrointestinal cancers and downregulated in leukaemia and lymphoma (Supplementary Fig. S7B, C). In clinical samples, we verified a tight correlation between the expression of KLF5 and *CCAT1*. These findings imply the coordinated expression of the two genes in CRC and possibly in other type of human cancers.

The *KLF5* enhancer region plays a biologically important role in cancer stem-like properties. Thus, even heterogenous deletion mutant clones exhibited a decrease in CSC markers such as *LGR5*, *BMI1*, *CD133*, and *CD44v9*, restored susceptibility to chemotherapy, and reduced sphere formation. This could be attributed to the downregulation of *KLF5* because the KLF5 inhibitor suppressed CSC-related gene expression in CRC cell lines (Supplementary Fig. S8A, B). Moreover, we and another group recently showed that the treatment of miR-4711-5p targeting to the 3’-UTR of *KLF5* mRNA or *KLF5*-siRNA attenuated the CSC properties in CRC cell lines [10, 11]. We also showed in this study that high *KLF5* expression in CRC clinical samples was correlated with poor prognosis, which is consistent with other studies [10]. In addition, studies reported that *CCAT1* stimulated symmetric division and self-renewal, which are hallmarks of CSCs in lung cancer, and that *CCAT1* is required for the maintenance of stemness, proliferation, migration and invasion of breast cancer stem cells [53, 54]. It was also reported that *BMI1*, which is one of the CSC-related genes, was decreased by the knockdown of *CCAT1* [55]. Because *CCAT1* expression was decreased in the deletion mutants of the *KLF5* enhancer, it is suggested that the *KLF5* enhancer may play a crucial role in the maintenance of cancer stemness by regulating KLF5 and *CCAT1*. Taken together, these results show that targeting therapeutics against the *KLF5* enhancer, e.g., blockade of *KLF5* promoter–enhancer binding by the decoy oligonucleotide strategy may be an efficient therapeutic option for CSCs.

In summary, we identified an enhancer of *KLF5* downstream of the *KLF5* gene that interacted with the *KLF5* promoter, and this enhancer region was associated with CSC properties in CRC. We also found that the *KLF5* promoter interacted with the *CCAT1* TSS region, which is located on different chromosomes. Notably, our results suggest that the core-regulatory circuitry containing the *KLF5* gene product and co-factors help to construct three-dimensional genome interaction and regulates the gene expression of *KLF5* and *CCAT1* (Fig. 6d), and this mechanism may facilitate the maintenance of CSC properties in CRC.

### Reporting summary

Further information on research design is available in the Nature Research Reporting Summary linked to this article.

## Supplementary information

Supplemental material

Reporting Summary

## Data Availability

The ChIP-Atlas (http://chip-atlas.org/) database was used for the analysis of ChIP-seq and DNase-seq data. The following datasets were analysed in this study: GEO ID: GSM1890734, GSM2400470, GSM1890736 and GSM1250899. For the analysis of mRNA expression in the multiple types of cancer cell lines, the CCLE (The Cancer Cell Line Encyclopedia) database (https://portals.broadinstitute.org/ccle) was used. The accession number of the in vitro enChIP-seq data in this study is DRA007368.
