# Supplementary material for: A stem cell marker KLF5 regulates CCAT1 via three-dimensional genome structure in colorectal cancer cells
Source: Br J Cancer. 2021 Oct 27;126(1):109–19. doi: 10.1038/s41416-021-01579-4 (PMC8727571; doi:10.1038/s41416-021-01579-4)
Supplement: Supplementary file 1 — Supplemental material [file 41416_2021_1579_MOESM1_ESM.pdf]

Supplementary Figure S1

A

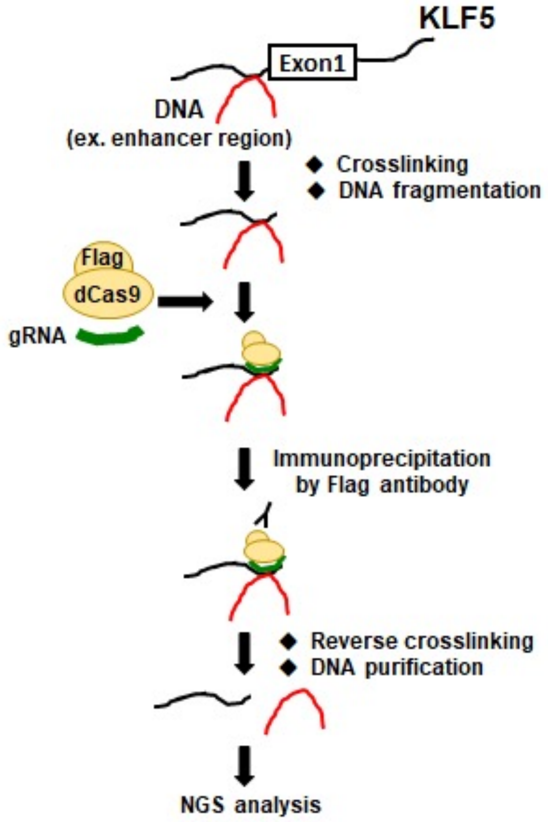

B

```
                gRNA-A
-600 TTGTTAAACGGATACACTCACTGCTAAAGATACACAGGGACAAGAGCTGT
     TTTAAATTTGTTTATCACACCGCCCCTAGTTTTCTGCTAAAGAGAAGTAC
-500 ATTTTGGATATTTAAAAGTACACACTCATTAGACAAATCTCTAAAAGCCT
                    gRNA-B
     CAGCGCCCCTACCTAGCTGCCTTCTGGGTTTCAGCTTTTGAGTTTCAGGT
-400 CCTTGCACAACACCTAAGACGTGCAATACTCTTTTTCTTCCTAATCCTGG
     CAGAGAACAGTGCGAAGAGATCTCCCTCCGCGTCTCCACCCTAATTCCCC
-300 CTCTTCCCAGATTCCACCCGCTCCGCTACTTCCCTCCGCCCCCGGCTCCG
     CCAGCCCAATCTGTCAGAGAAGTTGTGTACAAACTGCGCGGCCGCCCTGC
-200 GCGCGAAGCTCGTGGCCCGAGAGGGGTGCGGTCGGGCCGACGGAGGCGGG
     GCCCTGGCTGCCTCTCTCCCTGCTCATAGGCTGGCCGCTCAGGCCTGGCC
-100 GGCCTCGGGGCCTCGGGATTCGCGGCGGCGCTGCCAATCAGGCGATCGGG
     CCCCGCCCCCCCGGAGTTGGGTGAAATAGAGGCGGGCGTCAAGTGTCAGT
       KLF5
  +1 AGTCGCGGGGCAGGTACGTGCGCTCGCGGTTCTCTCGCGGAGGTCGGCGG
```

Supplementary Figure S1.
A, Schematic illustration of the *in vitro* enChIP method.
B, Genomic sequence of the *KLF5* promoter region. The -600~+50 region is shown. The *KLF5* TSS and the target sequences of gRNAs for *in vitro* enChIP-seq are indicated.

Supplementary Figure S2

A

	Chr	Start	End	Summit	*P* score	Closest Gene Symbol
	chr13	73632293	73632792	73632537		KLF5
#1	chr13	96216588	96216883	96216740	27.93	CLDN10
#2	chr13	63217092	63217370	63217195	25.33	
#3	chr13	74003796	74004073	74003995	21.73	
	chr13	109564198	109564464	109564333	21.29	MYO16
	chr13	62817225	62817434	62817298	20.08	
	chr13	35283237	35283456	35283332	19.36	
	chr13	74409399	74409617	74409485	19.32	KLF12
	chr13	76363009	76363245	76363123	19.32	LMO7
	chr13	40828199	40828409	40828327	18.54	
	chr13	34731119	34731391	34731293	18.48	
	chr13	107028728	107029019	107028866	17.36	LINC00460
	chr13	106622260	106622468	106622374	15.42	
	chr13	97846794	97847067	97846891	15.41	
	chr13	106095373	106095631	106095514	14.64	
	chr13	107002625	107002926	107002749	13.43	
	chr13	22396412	22396734	22396591	10.62	
	chr13	50422188	50422505	50422342	9.70	
	chr13	22237485	22237692	22237610	9.68	
	chr13	98283519	98283771	98283672	9.48	

B

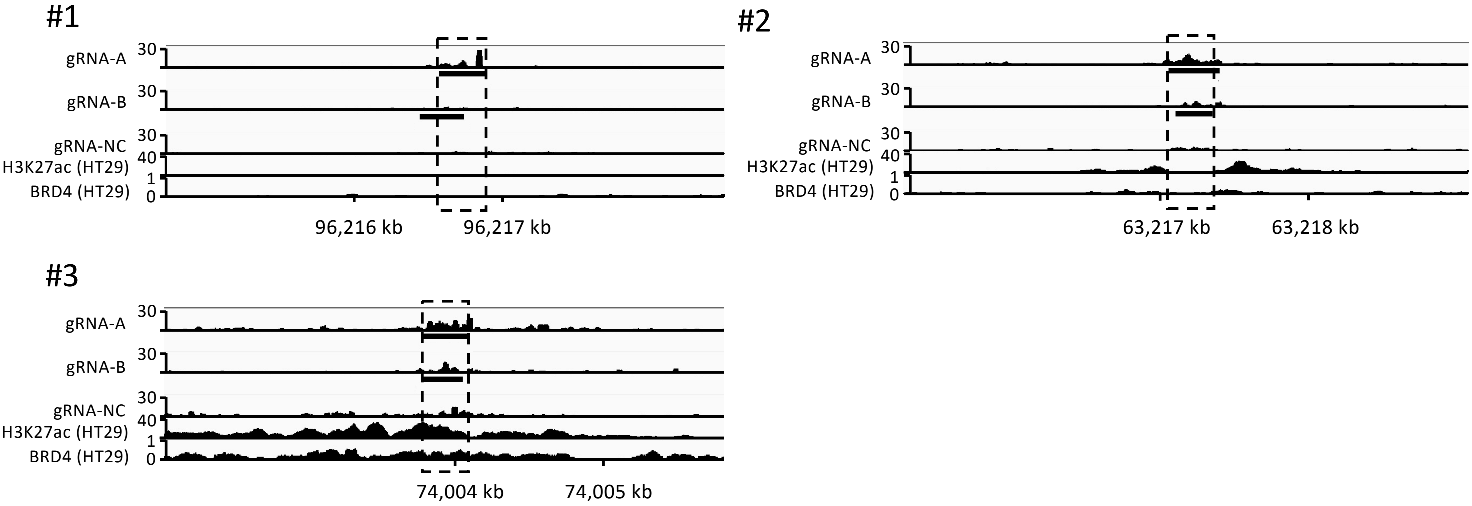

Supplementary Figure S2.

A, List of regions that bind to the *KLF5* promoter by *in vitro* enChIP-seq analysis. The regions of the top 19 highest *P* scores on chromosome 13 are shown. First line shows the KLF5 promoter region including the target region of two gRNAs.

B, IGV track of *in vitro* enChIP-seq peaks and ChIP-seq peaks of H3K27ac (HT29 colon cancer cell line) and BRD4 (HT29 colon cancer cell line) from the ChIP-Atlas database at the #1, #2, #3 region listed in S2A.

Supplementary Figure S3

A

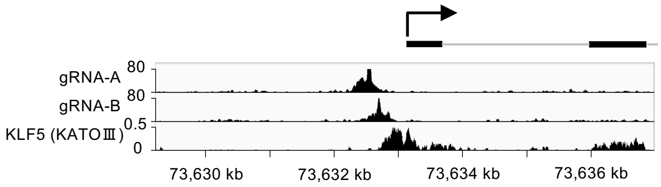

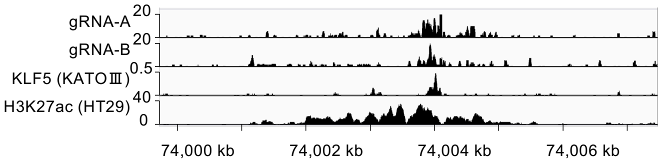

B

KLF5 promoter

Chr13: 73,632,543-73,633,142	
Name	Relative score
KLF15	1.000000005
KLF5	1.000000002
FOXL1	0.993649047
ZNF354C	0.991568145
MEIS1	0.991412464
GSX2	0.988713788
HOXC4	0.988177854
FOXG1	0.986265815
HOXD4	0.984911163
PITX1	0.981303179
RHOXF1	0.9811454
SP9	0.980423467
KLF16	0.978779943
FOXP3	0.978500055
HOXB3	0.978455587
HOXA2	0.977227913
RBPJ	0.977006343
ARNT::HIF1A	0.976049778
PDX1	0.975665978
SP3	0.97287621

KLF5 enhancer

Chr13: 74,003,945-74,004,045	
Name	Relative score
NFATC2	1.000000001
MZF1	0.999999979
SPIB	0.975072448
NFATC3	0.950526725
MZF1	0.947676606
SPIB	0.946799076
ZNF354C	0.930628178
BSX	0.928841033
SPIB	0.921871522
FOXD2	0.911041566
LBX1	0.908073129
MEIS1	0.908055285
TP73	0.889692841
NKX2-8	0.887035024
THAP1	0.880695953
MZF1	0.88062981
RHOXF1	0.880219168
RHOXF1	0.880219168
FIGLA	0.879176733
KLF5	0.877911246

C

KLF5

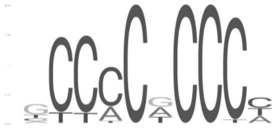

KLF5 promoter

chr13: 73,633,043-73,633,142

GGCCTCGGGGCCTCGGGATTCGCGGCGGCGCTGCCAAT
CAGGCGATCGG**GCCCCGCCCC**CCCGGAGTTGGGTGAAA
TAGAGGCGGGCGTCAAGTGTCAGT

KLF5 enhancer

chr13: 74,003,945-74,004,045

CTTTCTTCCCCTATTTTCACCTCTTCCCCAAAGCTTTGCTG
GGTAACTGGAAAAAAGGTGATTTCTTCCTCA**ACCACTCCT**
TGTCTGAACATGCAGCAATG

MEIS1

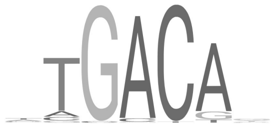

KLF5 promoter

chr13: 73,632,836-73,632,935

ATTCCCCCTCTTCCCAGATTCCACCCGCTCCGCTACTTCC
CTCCGCCCCCGGCTCCGCCAGCCCAAT**CTGTCAG**AGAAG
TTGTGTACAAACTGCGCGGCC

KLF5 enhancer

chr13: 74,003,945-74,004,045

CTTTCTTCCCCTATTTTCACCTCTTCCCCAAAGCTTTGCTG
GGTAACTGGAAAAAAGGTGATTTCTTCCTCAACCACTCC**T**
TGTCTGAACATGCAGCAATG

RHOXF1

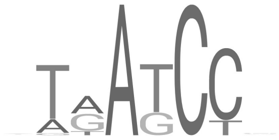

KLF5 promoter

chr13: 73,632,739-73,632,838

AGGTCCTTGCACAACACCTAAGACGTGCAATACTCTTTTT
CTTC**CTAATCCT**GGCAGAGAACAGTGCGAAGAGATCTCC
CTCCGCGTCTCCACCCTAATT

KLF5 enhancer

chr13: 74,003,945-74,004,045

CTTTCTTCCCCTATTTTCACCTCTTCCC**CAAAGCTT**TGCTG
GGTAACTGGAAAAAAGGTGATTTCTTCCTCAACCACTCCT
TGTCTGAACATGCAGCAATG

ZNF354C

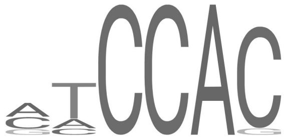

KLF5 promoter

chr13: 73,632,785-73,632,884

AATCCTGGCAGAGAACAGTGCGAAGAGATCTCCCTCCGC
GT**CTCCAC**CCTAATTCCCCCTCTTCCCAGATTCCACCCGC
TCCGCTACTTCCCTCCGCCCC

KLF5 enhancer

chr13: 74,003,945-74,004,045

CTTTCTTCCCCTATTTTCACCTCTTCCCCAAAGCTTTGCTG
GGTAACTGGAAAAAAGGTGATTTCTTCCTC**AACCAC**TCCT
TGTCTGAACATGCAGCAATG

Supplementary Figure S3 continued

D

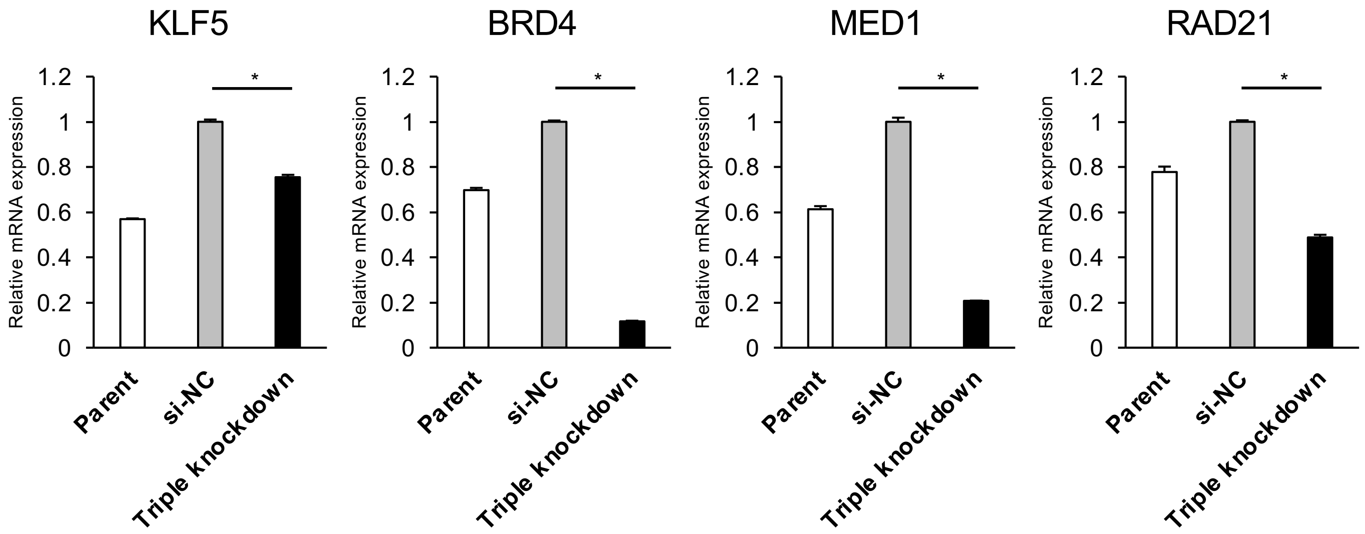

E

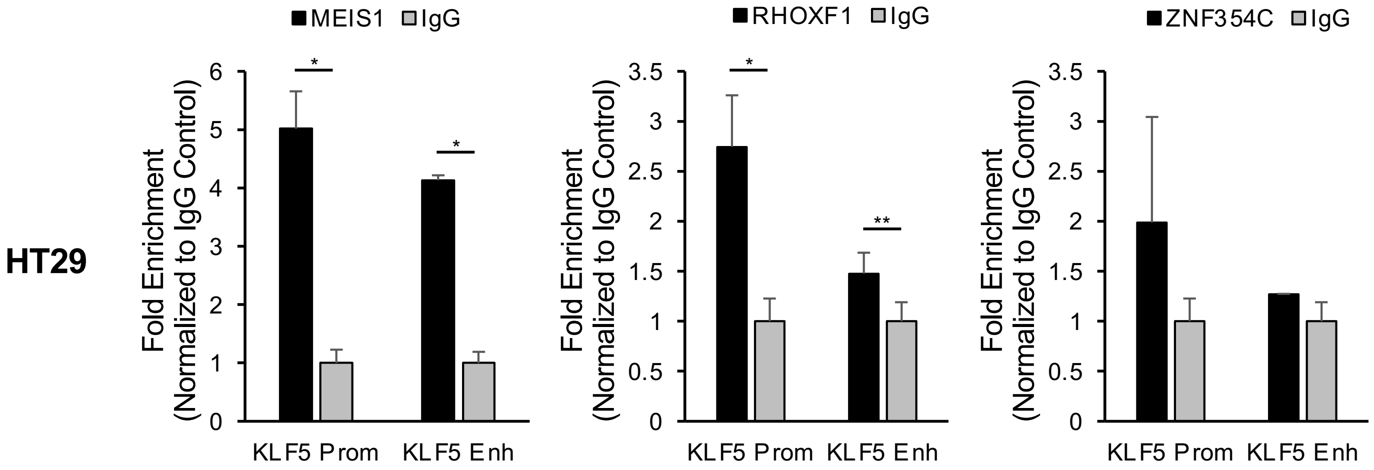

Supplementary Figure S3.

A, IGV track of in vitro enChIP-seq peaks and ChIP-seq peaks of KLF5 (KATO III gastric cancer cell line) and H3K27ac (HT29 colon cancer cell line) from the ChIP-Atlas database at the *KLF5* promoter (top) and enhancer (bottom).

B, List of transcription factors that may bind to the *KLF5* promoter (left) and *KLF5* enhancer (right) predicted by the JASPAR core transcription factor database.

C, Sequence of the *KLF5* promoter and enhancer region. The predicted KLF5, MEIS1, RHOXF1, ZNF354C binding sequence is shown in bold and underlined. The consensus sequence for KLF5, MEIS1, RHOXF1, ZNF354C is also shown at the top.

D, Expression level of *KLF5* mRNA in triple knockdown of *BRD4*, *MED1* and *RAD21* in HT29 cells (30nM for each). The relative value is calculated by the expression level of the si-negative control transfected cells (siNC). *P < 0.01

E, The binding of the MEIS1, RHOXF1, ZNF354C proteins to the *KLF5* promoter and enhancer in HT29 cell lines analyzed by ChIP-qPCR. Fold enrichment normalized to the value of the IgG control is shown. *P < 0.01, **P < 0.05

Supplementary Figure S4

A

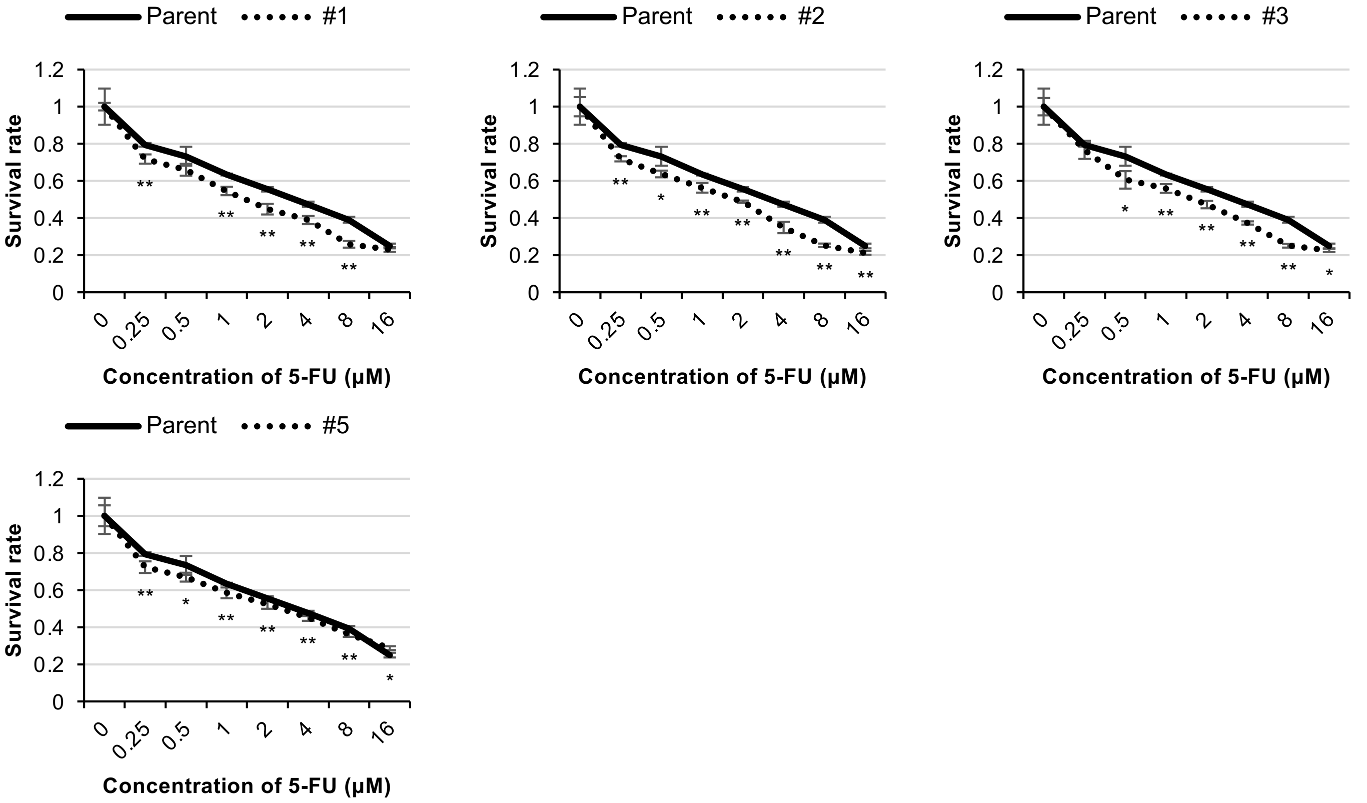

B

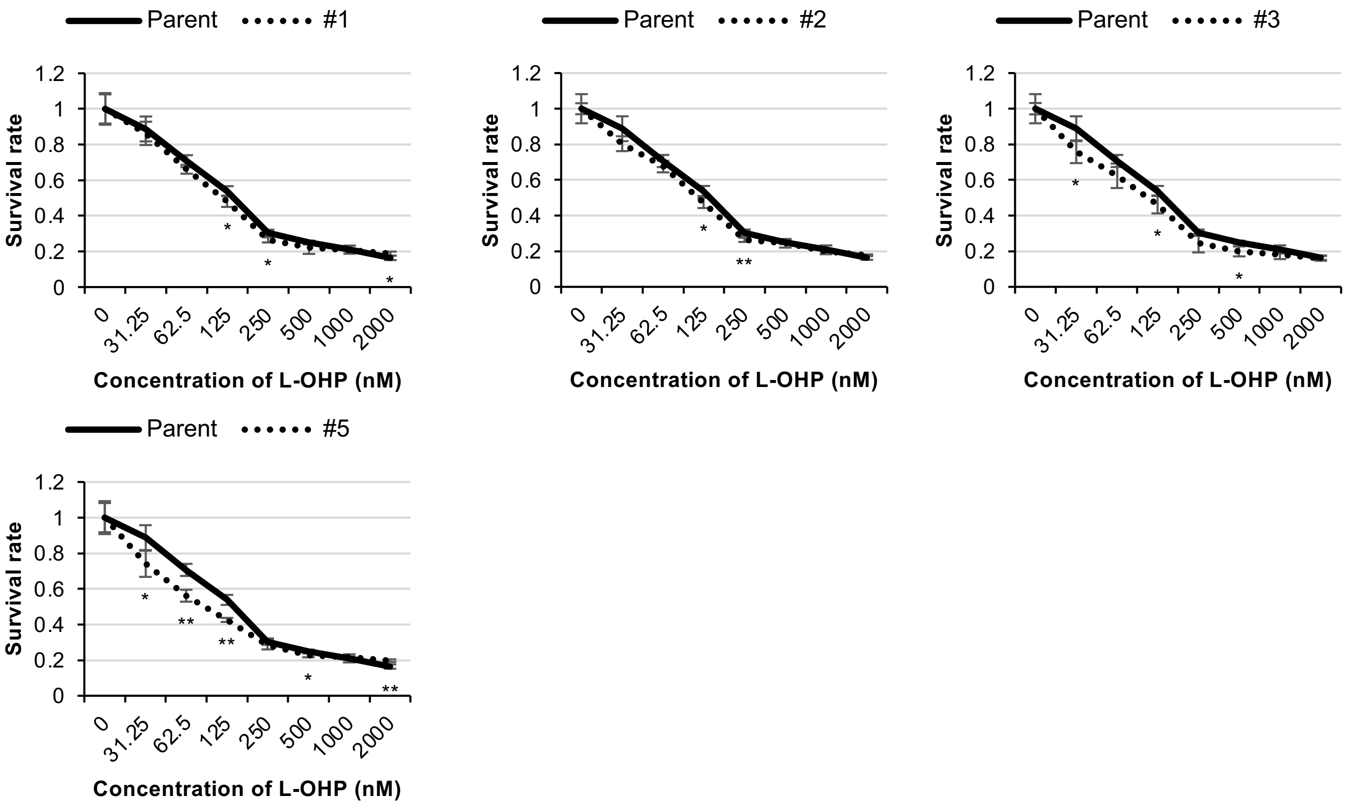

Supplementary Figure S4

C

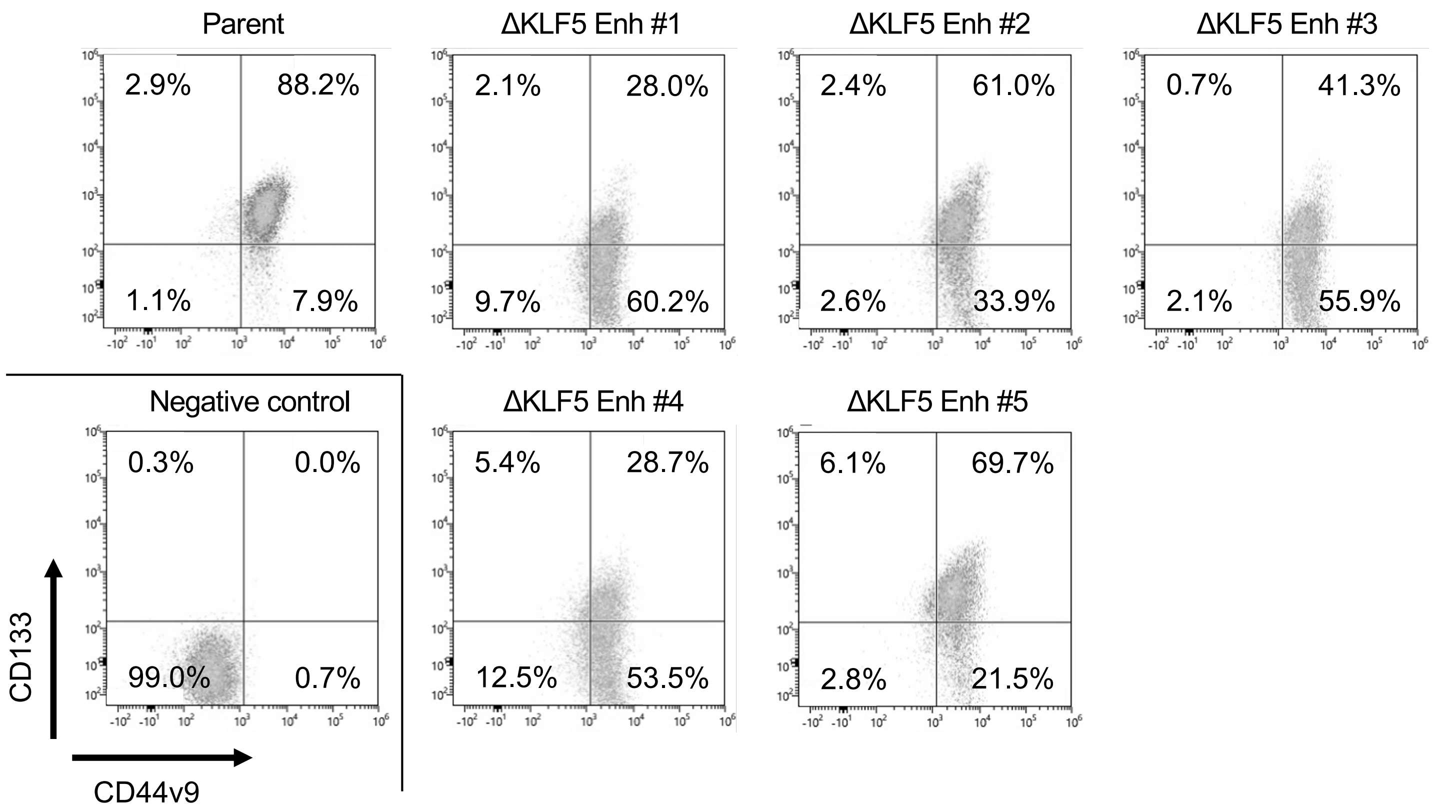

Supplementary Figure S4.

A, B, The survival rate of parental cells and heterodeletion mutants (#1, #2, #3, #5) of the *KLF5* enhancer after 5-FU (A) and L-OHP (B) treatment. **P* < 0.05, ***P* < 0.01

C, The expression levels of CD44v9 and CD133 analyzed by flow cytometry in parental cells and five heterodeletion mutants of the *KLF5* enhancer. X-axis shows the level of CD44v9 and Y-axis shows the level of CD133.

Supplementary Figure S5

A

	Chr	Start	End	Summit	*P* score	Closest Gene Symbol
	chr13	73632293	73632792	73632537		KLF5
#1	chr8	128231146	128231713	128231437	74.95	CCAT1
#2	chr8	130709462	130709732	130709607	50.52	
#3	chr5	90245615	90245941	90245779	43.36	GPR98
	chr8	134664222	134664538	134664399	40.78	
	chr17	49031649	49031935	49031811	39.01	
	chr7	2393986	2394299	2394130	37.54	EIF3B
	chr12	97962427	97962687	97962565	37.10	
	chr4	102614998	102615464	102615153	37.07	
	chr1	149658502	149658727	149658650	36.44	
	chr14	53925052	53925398	53925185	34.86	
	chr1	214120596	214120931	214120768	33.43	PROX1-AS1
	chr5	55652031	55652694	55652327	32.90	
	chr2	47859220	47859432	47859338	32.04	
	chr15	96596976	96597236	96597114	31.67	LOC101927214
	chr17	28113185	28113450	28113316	31.34	SSH2
	chr20	33349872	33350195	33349941	30.93	NCOA6
	chr1	59050230	59050494	59050360	30.80	
	chr12	27763451	27763788	27763631	30.80	PPFIBP1
	chr7	30266480	30266755	30266622	30.55	

B

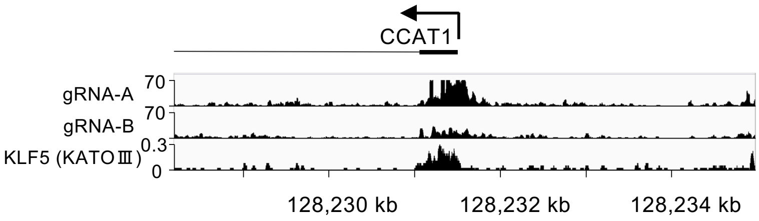

C

CCAT1 TSS

Chr8: 128,231,387-128,231,487	
Name	Relative score
NKX6-2	0.966311436
NKX6-1	0.962605988
TEAD3	0.953317826
HOXB3	0.946577994
PDX1	0.945168664
NOTO	0.944179531
KLF5	0.944020619
HOXB2	0.943819501
TEAD1	0.94012041
FOXP3	0.935091911
VAX2	0.930021978
HOXA2	0.929447423
BSX	0.929327814
VAX1	0.92804524
TEAD4	0.926144107
GSX1	0.919541727
GSX2	0.916499969
LHX9	0.915621454
MNX1	0.915284221
RHOXF1	0.914474309

KLF5

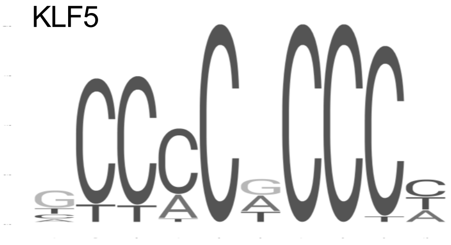

chr8: 128,231,387-128,231,487

AATCTAGAAGA**AGGGAGTGGG**TGAAGAATC
CCATTTGTAAAGAGGATAGCAGGTTAATGAA
AAGAAGCAGAGGTATGCGTAGGTGATAGTG
TTTCTACAAG

D

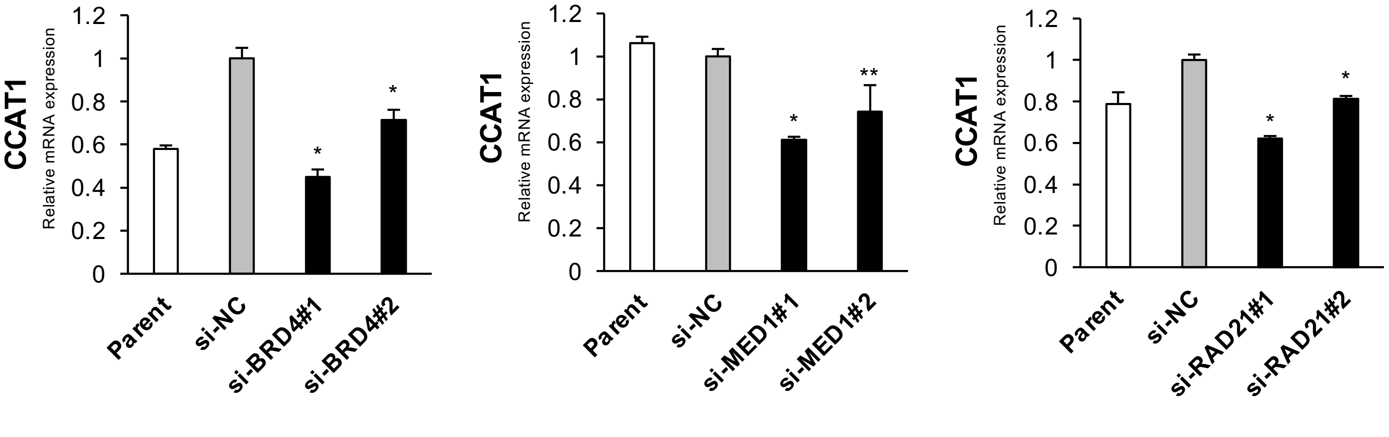

Supplementary Figure S5.

A, List of regions that bind to the *KLF5* promoter by analyzing the *in vitro* enChIP-seq data. The regions of the top 19 highest P scores in all chromosomes are shown. First line shows the KLF5 promoter region including the target region of two gRNAs.

B, IGV track of *in vitro* enChIP-seq peaks and ChIP-seq peak of KLF5 (KATO III gastric cancer cell line) in the *CCAT1* TSS region from the ChIP-Atlas database.

C, List of transcription factors that may bind to the *CCAT1* TSS region predicted by the JASPAR core transcription factor database. The sequence of the *CCAT1* TSS region is also shown. The predicted KLF5 binding sequence is shown in bold and underlined. The consensus sequence for KLF5 is also shown.

D, Expression level of *CCAT1* RNA in HT29 cells transfected with siRNAs against *BRD4*, *MED1* and *RAD21*. The relative value is calculated by the expression level of the si-negative control transfected cells (siNC). *P < 0.01, **P < 0.05

Supplementary Figure S6

CCAT1 Expression

KLF5 Expression	Weak	Moderate	Strong	Total
Weak	0 (0.0 %)	1 (5.9 %)	0 (0.0 %)	1 (5.9 %)
Moderate	0 (0.0 %)	6 (35.3 %)	1 (5.9 %)	7 (41.2 %)
Strong	0 (0.0 %)	2 (11.8 %)	7 (41.2 %)	9 (52.9 %)
Total	0 (0.0 %)	9 (52.9 %)	8 (47.1 %)	17

$P = 0.0256$

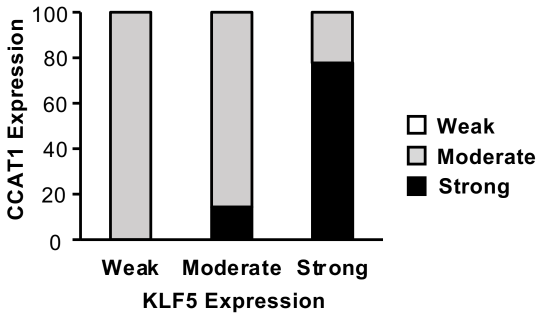

Supplementary Figure S6.
The percentage of each score (weak, moderate, strong) of KLF5 protein and *CCAT1* RNA expression in well differentiated CRC clinical samples. The bar chart is also shown.

Supplementary Figure S7

A

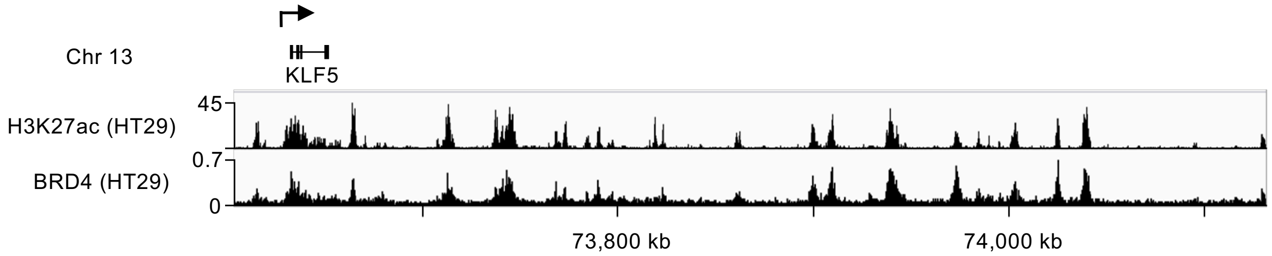

B

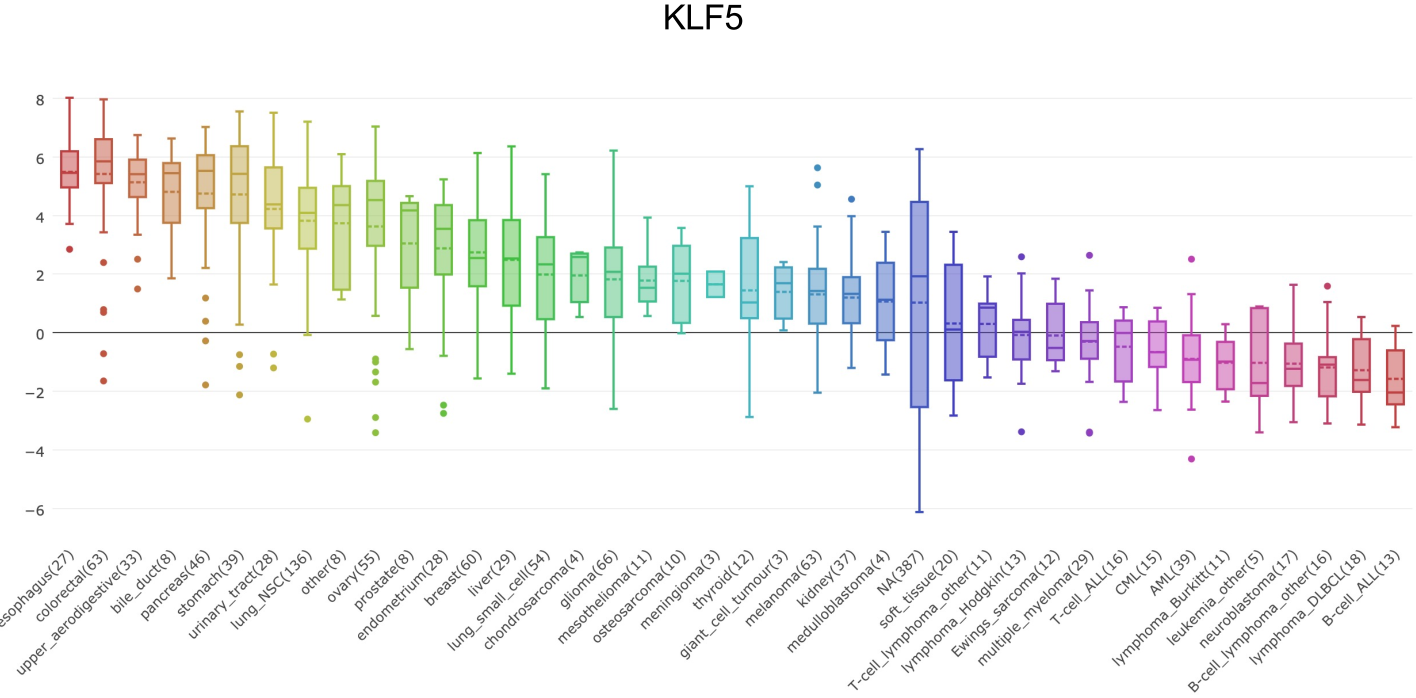

C

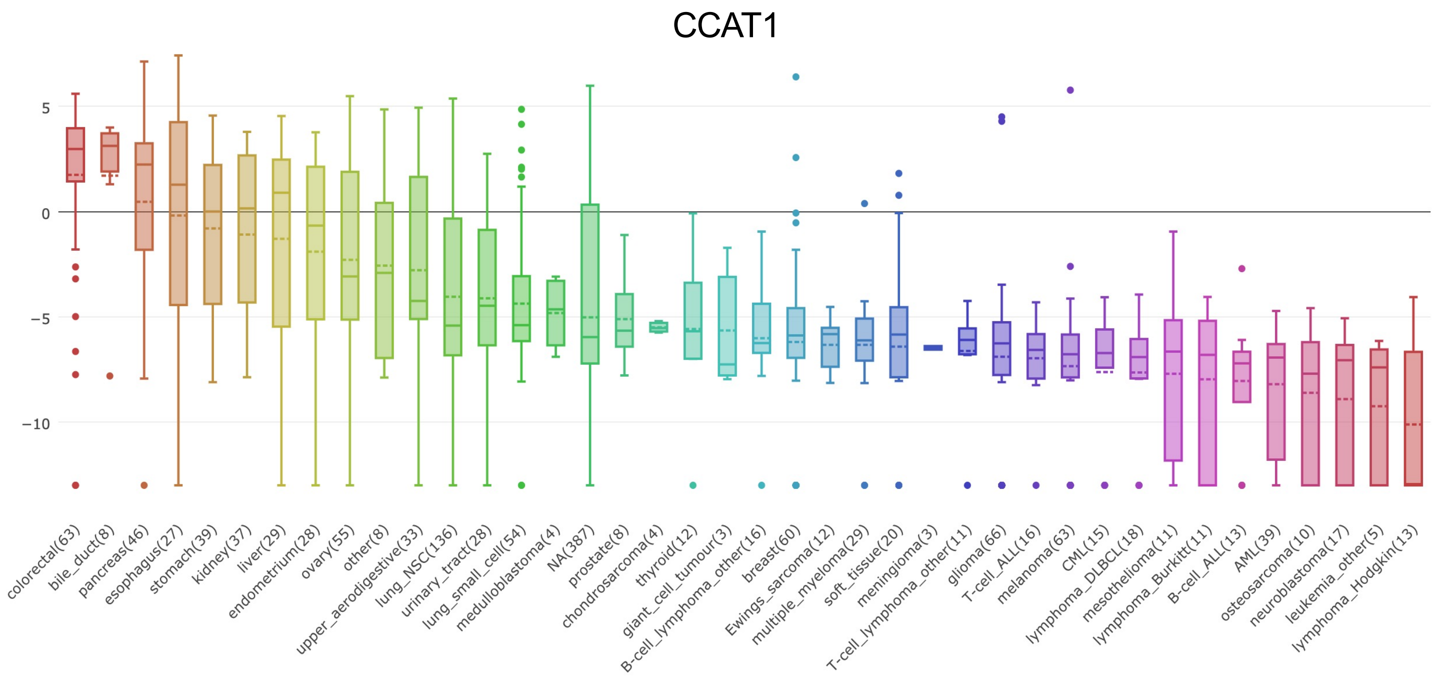

Supplementary Figure S7.
A, IGV track of ChIP-seq peaks of H3K27ac and BRD4 from the ChIP-Atlas database downstream of the *KLF5* gene in HT29 cells.
B, *KLF5* mRNA expression levels in many types of cancer cell lines from the CCLE database. The digit after the name of cancer type indicates the number of cell lines.
C, *CCAT1* RNA expression in many types of cancer cell lines from the CCLE database. The digit after the name of cancer type indicates the number of cell lines.

Supplementary Figure S8

A

HT29

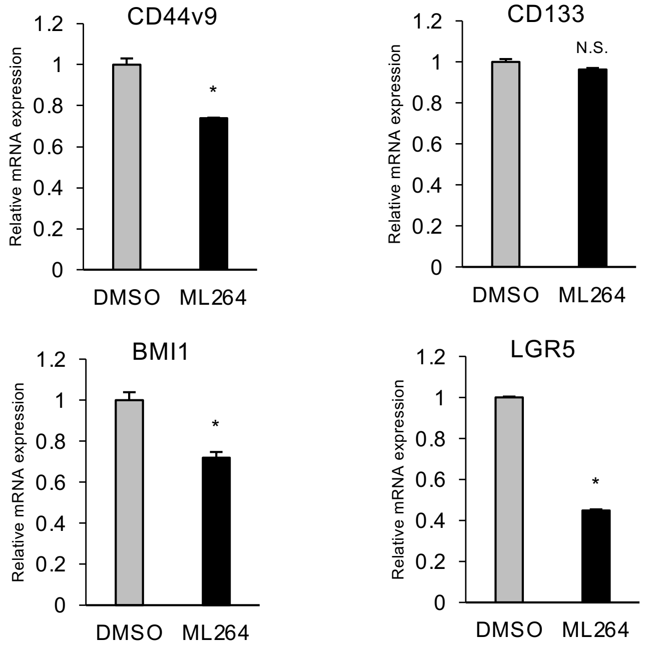

B

SW48

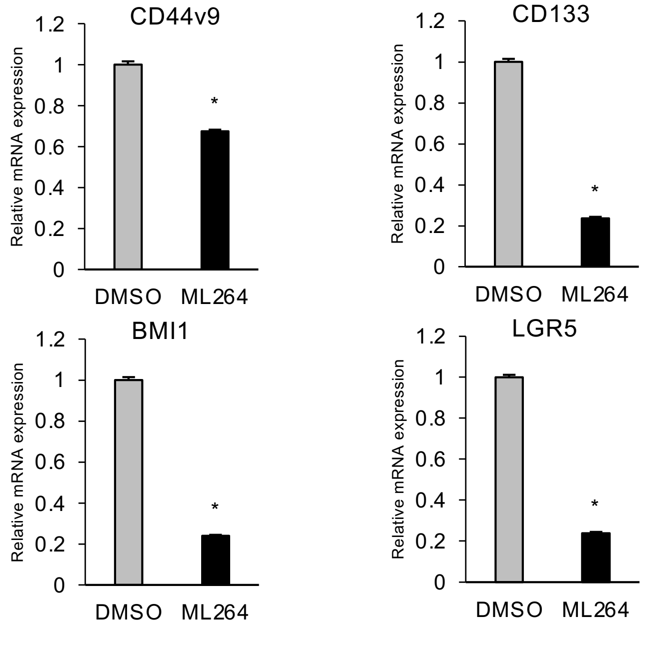

Supplementary Figure S8.
Expression level of CSC-related genes in ML264-treated cells (HT29 **(A),** SW48 **(B)**). ML264 was treated for 24 hours. The relative value is calculated by the expression level of DMSO-treated control cells. *$P < 0.01$, N.S. = Not significant

Supplementary Table S1

Primer sequnece for qRT-PCR	
KLF5_F	CCACCACCCTGCCAGTTAAC
KLF5_R	TAAACTTTTGTGCAACCAGGGTAA
GAPDH_F	CAACTACATGGTTTACATGTTC
GAPDH_R	GCCAGTGGACTCCACGAC
CCAT1_F	AGAAACACTATCACCTACGC
CCAT1_R	CTTAACAGGGCATTGCTAATCT
BMI1_F	TGTAAAACGTGTATTGTTCGTTAC
BMI1_R	CAATATCTTGGAGAGTTTTATCTGACC
LGR5_F	CCCTTCATTCAGTGCAGTGTT
LGR5_R	AGCAGGTGTTCACAGGGTTT
CD44v9_F	ATAATGAGCTTCTCTACATCACATGAAGGC
CD44v9_R	ATAATGCAGAGTAGAAGTTGTTGGATGGT
CD133_F	TGACAAGCCCATCACAACAT
CD133_R	CGCCTGAGTCACTACGTTGC
BRD4_F	ACCAGTTTGCATGGCCTTT
BRD4_R	TTGTTTTCCAAGCGCTTCTT
MED1_F	CTGGAACGGCTCCATGCAA
MED1_R	CTTCTCCATGACTTGACGCAC
RAD21_F	GGATAAGAAGCTAACCAAAGCCC
RAD21_R	CTCCCAGTAAGAGATGTCCTGAT

siRNA sequence (5' - 3' sense sequence)	
NC siRNA	AUCCGCGCGAUAGUACGUA
BRD4 siRNA#1	CGUCCGAUUGAUGUUCUCCAA
BRD4 siRNA#2	CCUGGAGAUGACAUAGUCUUA
MED1 siRNA#1	GCUGGUCCCUUGGAUAAGA
MED1 siRNA#2	CCAGUACAGGUGGAUCUAA
RAD21 siRNA#1	GAUGACGACAUGUUAGUAA
RAD21 siRNA#2	GGAGCCUAUUGAUAUAACU
KLF5 siRNA#1	CGAUUACCCUGGUUGCACA
KLF5 siRNA#2	CUAUAAUUCCAGAGCAUAA
CCAT1 siRNA#1	GGAGGGUGCUUGACAAUAAUU
CCAT1 siRNA#2	GAGAAGACCAUAAGAAGAU
CCAT1 siRNA#3	GGAUGGACAUCAGAACUAU

Supplementary Table S1 continued

Primer sequnece for ChIP-qPCR	
KLF5 Promoter ChIP_F	CTCTCTCCCTGCTCATAGGC
KLF5 Promoter ChIP_R	CCGCGACTACTGACACTTGA
KLF5 Enhancer ChIP_F	CTGAGCTAGGCCCCTTTCTT
KLF5 Enhancer ChIP_R	GCTGCATGTTCAGACAAGGA
CCAT1 TSS ChIP_F	GGGCTTATTGTTGGGACAGA
CCAT1 TSS ChIP_R	CGCTCACGATTCACAGAAAA

Oligonucleotide sequence for generation of deletion mutant	
KLF5 Enh Left_sense	CACCGGTTTGTGATCGGGTGATCCC
KLF5 Enh Left_antisense	AAACGGGATCACCCGATCACAAACC
KLF5 Enh Right_sense	CACCGTGAGGGCTCGGTATATTCTT
KLF5 Enh Right_antisense	AAACAAGAATATACCGAGCCCTCAC
CCAT1 TSS Left_sense	CACCGAAATTGATCTATCGGACCAA
CCAT1 TSS Left_antisense	AAACTTGGTCCGATAGATCAATTTC
CCAT1 TSS Right_sense	CACCGCAATTAGAAGTAGATCCGTA
CCAT1 TSS Right_antisense	AAACTACGGATCTACTTCTAATTGC

Primer sequence for validation of deletion muttant	
KLF5 Enh-F1	TCCTAAGCTCTGGCTTCTGC
KLF5 Enh-R1	GCCATGAGTGTCAGAATTGC
KLF5 Enh-R2	TCATCCCTGTCACCAGATCA
CCAT1-F1	CGTCCAACAATGCACAGAAC
CCAT1-R1	CCATTCAGTCCTCAGGGACA
CCAT1-R2	CTTGGCTTTGGCTTGTCATT

gRNA sequence	
crRNA-A (KLF5 promoter)	GUGAGUGUAUCCGUUUAACAguuuuagagcuaugcuguuuug
crRNA-B (KLF5 promoter)	CCCAGAAGGCAGCUAGGUAGguuuuagagcuaugcuguuuug
crRNA-NC (chicken Pax5)	CGGCAGGCUCGGGUGCGCCUguuuuagagcuaugcuguuuug
tracrRNA	AAACAGCAUAGCAAGUUAAAAUAAGGCUAGUCCGUUAUCAACUUGAAAAAGUGGCACCGAGUCGGUGCU

Supplementary Table S1.
List of primers, siRNA, oligonucleotides and gRNAs.

Supplementary Table S2

Charactearistics	Total n = 131	KLF^{LOW} n =66	KLF^{HIGH} n =65	P-value
Male/female	72/59	32/34	40/25	0.1611
Location, Right/Left	42/89	17/49	25/40	0.1369
CEA (ng/mL), median	5.1 (0.9-10943)	4.7 (1.0-233.7)	7.3 (0.9-10943)	0.0690
CA19-9 (U/mL), median	13 (0-22358)	13 (0-889)	12 (0-22358)	0.1815
Histologic type (tub/por・muc・sig)	127/4	65/1	62/3	0.3653
Lymph invasion (-/+)	35/96	18/48	17/48	1.0000
Venous invasion (-/+)	50/81	26/40	24/41	0.8577
Tumor invasion (T0-3/T4)	63/68	34/32	29/36	0.4859
Lymph node metastasis (-/+)	64/67	28/38	36/29	0.1634
Curability (curative/non-curative)	114/17	61/5	53/12	0.0736

Supplementary Table S2.
Comparison of patients' background stratified by *KLF5* mRNA expression in CRC clinical samples. No significant differences are observed between two groups.
